# The Role of Self-Efficacy and Injunctive Norms in Helping Older Adults Decide to Stay Home During the COVID-19 Pandemic

**DOI:** 10.3389/fpubh.2021.660813

**Published:** 2021-06-04

**Authors:** Jonathan T. Macy, Christopher Owens, Kristina Mullis, Susan E. Middlestadt

**Affiliations:** ^1^Department of Applied Health Science, School of Public Health, Indiana University, Bloomington, IN, United States; ^2^Institute for Sexual and Gender Minority Health and Wellbeing, Northwestern University, Chicago, IL, United States

**Keywords:** older people, stay at home orders, reasoned action approach, self-efficacy, belief determinants, COVID-19

## Abstract

**Purpose:** Because older adults are at elevated risk of COVID-19-related adverse health outcomes, and staying at home is an effective strategy to avoid unnecessary exposures, the current formative study used the Reasoned Action Approach (RAA) to identify the beliefs underlying older adults' decision to stay home for the next month.

**Methods:** The participants (weighted *n* = 206, age 65-94) for the current study were selected from a nationally representative online survey of US adults from April 10-20, 2020. We used multiple linear regression to estimate the relative contribution of the four RAA global constructs (instrumental attitude, injunctive norms, descriptive norms, and self-efficacy) in explaining intention to stay home after controlling for demographic covariates. We also conducted a content analysis to identify beliefs about advantages, disadvantages, and facilitators of staying home.

**Results:** After controlling for demographic characteristics, injunctive norms (*b* = 0.208; SE = 0.059; *B* = 0.213, *p* < 0.01) and self-efficacy (*b* = 0.532; SE = 0.058; *B* = 0.537, *p* < 0.001) showed statistically significant independent associations with intention to stay home. The specific beliefs underlying the decision to stay home spanned across health and wellness dimensions and suggested interpersonal, mental health, and leisure/recreational facilitators.

**Conclusions:** These findings suggest three public health intervention targets. First, self-efficacy building interventions could enhance older adults' perceptions of their ability to stay home to avoid unnecessary exposures. Second, health communication messages to address injunctive norms could emphasize that people important to older adults think they should stay home. Third, for the youngest of the older adults, health communication messages could emphasize the advantages of staying home.

## Introduction

In response to the coronavirus disease 2019 (COVID-19) outbreak in the spring of 2020, governors across the US issued stay-at-home executive orders to prevent the spread of the virus and protect the health care infrastructure from becoming overwhelmed. Stay-at-home orders have been shown to be effective at preventing COVID-19 infections (1, 2), reducing hospitalizations related to COVID-19 (3), and lowering COVID-19-related deaths (4). Under these circumstances, it is especially important for older adults to stay home because they are disproportionately affected by COVID-related severe complications, hospitalization, and death compared to other age groups (5, 6). As the COVID-19 pandemic persists, protecting older adults will continue to be an important public health goal until mass vaccination results in herd immunity. Moreover, the lessons learned from the pandemic will inform the responses to future public health crises that threaten vulnerable groups such as older adults.

Strategies to promote engagement in a behavior such as staying home are more effective if they are based on evidence and theory (7). Therefore, theory-based formative research using a cross-sectional design to identify the beliefs underlying people's behavioral decisions is an essential first step to inform intervention design and testing. The Reasoned Action Approach (RAA) (8) and its predecessors, the Theory of Planned Behavior (9) and the Integrative Model (10), have been successfully applied to understand how people make decisions about many health behaviors (9, 11, 12). In addition, the RAA has been used to examine health behaviors and beliefs of older adults (13–15).

According to the RAA (8), intention is the belief factor most closely related to behavior. Intention in turn is associated with three global theoretical constructs: attitude toward the act, perceived norms, and perceived behavioral control. Attitude toward the act represents individuals' evaluation of the action of staying home. It includes an instrumental component that is related to outcome expectations and an experiential component that is related to affect. In the current study, we assessed the instrumental component of attitude. Perceived norms assess social pressure to engage in a target behavior and also include two components. The injunctive component reflects what people perceive that those important to them expect them to do, and the descriptive component reflects what people perceive other people like them will do. Finally, perceived behavioral control represents the extent to which people perceive that they are capable of performing the behavior. In this study, we focused on the self-efficacy component of perceived behavioral control. Self-efficacy reflects the extent to which individuals perceive that they have the capacity to carry out the behavior in question.

Underlying these global constructs are beliefs that can be identified *via* a qualitative elicitation. Of relevance here, beliefs about the advantages and disadvantages perceived about engaging in the action underly the attitude toward the act of staying home, and beliefs about the circumstances that might facilitate the behavior underly perceived behavior control related to staying home. However, not all beliefs listed by a priority group operate as salient. Thus, an important step in applying the RAA is to conduct a salient belief elicitation to identify the outcomes and circumstances that are at the top-of-the-mind of the specific priority group (16).

While there has been some research (17) on people's beliefs about and support for staying home, to our knowledge, there is only one published study (18) to date that examined the beliefs associated with intention or behavior related to staying at home specifically among older adults. In that study, attitude and subjective norms were associated with intention to socially isolate after Maryland's stay-at-home order was lifted (18). Although an important first contribution to the literature, that study relied on a convenience sample of older adults living in one US state. The current study capitalized on data obtained from a nationally representative sample of US adults to test the association between four RAA global constructs and intention to stay home for one key segment of the US population, older adults. These relationships were tested over and above the contributions of demographic factors. We hypothesized that the four global constructs would explain a large percent of the variation in intention, one that was statistically significantly larger than the variation explained by the demographic characteristics. In addition, because of the relatively large age span that includes older adults (65-94 in the current study), we tested for moderating effects of age on the relationships between the four global constructs and intention to stay home.

Furthermore, we conducted a salient belief elicitation to identify the advantages and disadvantages underlying attitude, and the facilitators and barriers underlying self-efficacy. The goal of both analyses was to identify beliefs that might be operating as drivers of intention. Currently, the literature lacks empirical evidence of what older adults believe about staying home. With a better understanding of beliefs associated with intention, public health professionals can design behavioral interventions that focus on the global constructs that explain the most variation in intention to engage in the behavior and address the specific beliefs underlying these global factors (e.g., what might make it easier for older adults to stay home). This is particularly important when a priority group is at elevated risk for serious outcomes, as is the case with older adults and COVID-19.

## Materials and Methods

### Study Participants

Data for the current study represent a subset of data that were obtained from a nationally representative online survey of US adults (age 18+ years) from April 10-20, 2020. Participants were recruited into the larger study using the Ipsos Knowledge Panel, a nationally representative, probability-based sample established using address-based sampling *via* the US Postal Service's Delivery Sequence File. These surveys have been shown to generate high-quality and generalizable results (19). Ipsos provides a web-enabled device and free Internet service to households without an Internet connection to ensure all panel members can access surveys. Using an equal probability selection method, members of the panel were sampled and invited to participate in the survey. Sampled participants were emailed an invitation and link to the online survey. Ipsos maintains an incentive program for participation in individual surveys, including drawings for prizes and cash rewards. Ethical approval for the study protocol was provided by the Indiana University Human Subjects Office (#2004194314).

Of the 1,632 Knowledge Panel members invited to participate in the larger study, 1,010 (61.9%) completed the survey. For this study, we selected participants age 65 and over (*n* = 273). We excluded 13 (4.8%) participants who were missing data resulting in a final unweighted sample of 260 eligible for analyses. A general population weight (calculated and provided by Ipsos) was applied to the data to minimize bias and variance due to non-response error. Weighting was calculated based on the latest March supplement of the Current Population Survey (CPS) with variables such as gender, race/ethnicity, age, education, census region, and household income. The final weighted sample size was 206.

### Measures

#### Demographic Characteristics

Study participants reported their age, sex, race/ethnicity, marital status, highest level of education completed, income, employment status, and political ideology. For analyses, education was dichotomized into less than a bachelor's degree vs. bachelor's degree or higher, and income was dichotomized into < $75,000 vs. $75,000 or more. Political ideology was assessed on a seven-point scale from extremely liberal to extremely conservative using an item from the General Social Survey (20).

#### Close-Ended RAA Questions

We first defined the behavior of interest with the statement “Many of us have been told to stay home, which means to stay in your house or apartment EXCEPT to get food, care for a relative or friend, get necessary health care, go to an essential job, or exercise separated from others.” Then we assessed five RAA constructs, with measures based on operationalization recommendations of the RAA developers (8). Participants were asked how much they agreed or disagreed (1 = strongly disagree, 5 = strongly agree) with a set of statements about “staying home for the next month.” *Intention* was assessed with the statement, “I plan to stay home for the next month.” Because of space and time limitations of the survey, we were limited to four belief predictors. For attitude toward the act, we selected good-bad to represent the *instrumental component of attitude* with the statement, “My staying home for the next month is a good thing to do.” We assessed both components of perceived norms. For *injunctive norms*, we used the statement, “Most people important to me think I should stay home for the next month.” For *descriptive norms*, we used the statement, “Most people like me will stay home for the next month.” We assessed the *capacity component of perceived behavioral control* with an item that is similar to the construct of self-efficacy, “I am confident that I can stay home for the next month.”

#### Open-Ended RAA Questions

To identify the salient advantages underlying attitude, participants were asked to “Name one good thing that might happen if you stay home for the next month.” To identify salient disadvantages underlying attitude, participants were asked to “Name one bad thing that might happen if you stay home for the next month.” Finally, to identify the salient facilitators underlying perceived behavioral control, participants were asked to “Name one thing that might make it easier for you to stay home for the next month.”

### Analysis

#### Statistical Analysis

Analyses were conducted using SPSS version 25.0 (21). We used multiple linear regression to estimate the relative contribution of the four global constructs in explaining intention to stay home. The demographic factors age, sex, education, income, and political ideology were entered in the first block. We then entered the four RAA global constructs in the second block to test their contribution over and above demographic factors. Finally, to test for moderating effects of age on the relationships between the four global constructs and intention to stay home, we entered the two-way interactions between age and the four global constructs in the third block. A median split was used to create two age groups, 65-71 [*n* = 109 (53.0%)] and 72-94 [*n* = 97 (47.0%)]. The values for age and the four global constructs were mean-centered before computing the interaction terms.

#### Content Analysis

To identify the underlying beliefs, we conducted a multi-step inductive content analysis (16). The responses were loaded into an Excel file. Spanish responses were translated to English. A codebook was created with narrow codes by the second, third, and fourth authors. All of the responses were coded by the second author. To assess interrater reliability, 15% of the responses were coded by the third author, resulting in Kappas of 0.98 for advantages, 0.99 for disadvantages, and 1.00 for facilitators. Based on a frequency analysis of the narrow codes, the second and last authors discussed and agreed on categories of codes, resulting in 8 advantages, 12 disadvantages, and 13 facilitators.

## Results

As shown in Table 1, the sample was 53.0% female and 77.3% non-Latinx White. The mean age was 72.56 (SD = 6.32; range 65-94). About two-thirds were married (64.6%), 65.1% had less than a bachelor's degree, and 53.5% reported income of <$75,000. The majority of this sample of older adults (78.7%) were retired. The mean value for political ideology was 4.38 (SD = 1.61; range 1 = extremely liberal to 7 = extremely conservative).

**Table 1:** Sample characteristics (Weighted *N* = 206).

	***N* (%)**	**Mean (SD)**
**Age**		72.56 (6.32)
**Sex**		
Female	109 (53.0)	
Male	97 (47.0)	
**Race/Ethnicity**		
Black or African American, non-Latinx	22 (10.5)	
White, non-Latinx	159 (77.3)	
More than one race, non-Latinx	4 (2.2)	
Other, non-Latinx	8 (4.1)	
Latinx	12 (6.0)	
**Marital status**		
Married	133 (64.6)	
Widowed	30 (14.5)	
Divorced or separated	29 (14.0)	
Never married	14 (6.6)	
Living with partner	1 (0.3)	
**Education**		
Less than bachelor's degree	134 (65.1)	
Bachelor's degree or higher	72 (34.9)	
**Income**		
< $75,000	110 (53.5)	
$75,000 or more	96 (46.5)	
**Employment status**		
Working—paid employee	22 (10.7)	
Working—self-employed	16 (7.7)	
Not working—retired	162 (78.7)	
Not working—other	6 (2.9)	
Political ideology		4.38 (1.61)
Intention to stay home		4.12 (0.96)
Instrumental attitude toward staying home		4.19 (0.94)
Injunctive normative belief about staying home		4.10 (0.98)
Descriptive normative belief about staying home		3.88 (0.91)
Self-efficacy to stay home		4.13 (0.97)

In terms of the RAA global constructs, the mean values were 4.12 (SD = 0.96) for intention, 4.19 (SD = 0.94) for attitude, 4.10 (SD = 0.98) for injunctive norms, 3.88 (SD = 0.91) for descriptive norms, and 4.13 (SD = 0.97) for self-efficacy. All constructs were measured on 1-5 scale. All four global constructs were significantly correlated with intention. The correlation with intention was highest for self-efficacy (*r* = 0.935, *p* < 0.01), next for injunctive norms (*r* = 0.731, *p* < 0.01), next for instrumental attitude (*r* = 0.699, *p* < 0.01), and lowest for descriptive norms (*r* = 0.647, *p* < 0.01).

Results from the regression analysis testing factors associated with intention to stay home are displayed in Table 2. Demographic factors were entered in the first block. Only political ideology was significantly associated with intention to stay home (*b* = −0.122; SE = 0.042 *B* = −0.205; *p* < 0.01). Participants who were more conservative had lower levels of intention to stay home than those who identified as liberal. After the RAA global constructs were added in the second block, there was a statistically significant increase in the adjusted R^2^, and political ideology was no longer significantly associated with intention. After controlling for demographic characteristics, injunctive norms (*b* = 0.208; SE = 0.059; *B* = 0.213, *p* < 0.01) and self-efficacy (*b* = 0.532; SE = 0.058; *B* = 0.537, *p* < 0.001) showed significant independent associations with intention to stay home. Neither attitude nor descriptive norms had significant weights.

**Table 2:** Results from multiple regression analysis testing factors associated with intention to stay home.

**Factor**	**Unstandardized coefficient**	**Standard error**	**Standardized beta coefficient**	***t***
**Model 1 (Adj R**^**2**^ **=** **0.042)**				
Age	0.017	0.010	0.115	1.674
Sex (0 = male; 1 = female)	0.181	0.133	0.094	1.367
Education (0 = less than bachelor's degree; 1 = bachelor's degree or higher)	0.030	0.153	0.015	0.193
Income (0 = < $75,000; 1 = $75,000 or more)	−0.148	0.142	−0.077	−1.040
Political ideology	−0.122	0.042	−0.205	−2.886^**^
**Model 2 (Adj R**^**2**^ **=** **0.732)**				
Age	0.0003	0.006	0.002	0.046
Sex (0 = male; 1 = female)	0.013	0.071	0.007	0.190
Education (0 = less than bachelor's degree; 1 = bachelor's degree or higher)	−0.040	0.082	−0.020	−0.486
Income (0 = < $75,000; 1 = $75,000 or more)	−0.149	0.075	−0.078	−1.975
Political ideology	−0.037	0.023	−0.063	−1.622
Instrumental attitude	0.106	0.061	0.103	1.728
Injunctive norms	0.208	0.059	0.213	3.512^**^
Descriptive norms	0.090	0.056	0.085	1.615
Self-efficacy	0.532	0.058	0.537	9.175^***^

** *p < 0.01;*

*** *p < 0.001*.

We also tested age by attitude, age by injunctive norms, age by descriptive norms, and age by self-efficacy by adding these interaction terms to the third block of the regression model. Only age by attitude was statistically significant (*b* = −0.253; SE = 0.122; *B* = −0.122, *p* = 0.04). To probe this significant interaction, we split the file into two groups, those age 65-71 and those age 72-94, and used multiple linear regression to test the association between attitude and intention controlling for demographics and the remaining RAA global constructs. These analyses showed that for the 65-71 year old group, attitude was significantly associated with intention to stay home (*b* = 0.264; SE = 0.078; *B* = 0.255, *p* = 0.001). However, attitude was not associated with intention for the 72-94 year old group (*b* = −0.015; SE = 0.094; *B* = −0.015, *p* = 0.877).

Table 3 shows the percent of older adults mentioning each of the eight advantages of staying home for the next month. By far, the most frequently mentioned benefit of staying home (41.3%) was an individual health benefit that staying home *might keep me healthy*. This category included responses such as I will not get COVID, I will not get sick, I will stay healthy, and I will not die. Few participants seemed to be concerned about the health of family members or other individuals. The second most frequently mentioned advantage, mentioned by just more than a quarter of the older adults (27.1%), was that staying home *might allow me to catch up on things at home*. This included responses such as catching up on chores and cleaning, doing home projects, and reading. The third most commonly cited advantage of staying home (10.6%) was the population health benefit that *my staying might slow or stop the spread of COVID-19*.

**Table 3:** Frequency of perceived advantages of staying home.

**My staying home for the next month…**	***N***	**%**
**Individual health benefits**		
Might keep me healthy	85	41.3
Might keep my family healthy	3	1.3
Might keep others healthy	3	1.7
**Population health benefits**		
Might slow or stop the spread of COVID-19	22	10.6
**Leisure and recreational benefits**		
Might allow me to catch up on things	56	27.1
**Interpersonal benefits**		
Might allow me to spend time with my family	8	3.9
**Financial benefit**		
Might help me save/spend less money	15	7.2

Table 4 shows the disadvantages of staying home that span a range of wellness dimensions, including physical, mental, interpersonal, financial, and occupational health. The most frequent disadvantage, mentioned by 16.9% of the older adults in our study, involved what might be seen as *temporary effects on mental or emotional well-being*. This included responses such as I might get bored, go stir crazy, or experience cabin fever. An additional 7.6% were concerned about more *serious mental health disadvantages*, including believing that staying home might lead to depression, anxiety, loneliness, or stress. The second most frequently mentioned disadvantage, mentioned by 15.3% of the older adults, involved *physical health problems*, such as gaining weight and exercising less. Our participants also saw interpersonal disadvantages of staying home. More specifically, 12.2% reported they *might miss interaction with friends and family*, including their own children and their grandchildren. Finally, 7.3% indicated they *miss getting out of the house to attend social events*. This included response about being confined to the house and not being able to attend recurring events like going out to dinner and one-time events like weddings.

**Table 4:** Frequency of perceived disadvantages of staying home.

**My staying home for the next month…**	**N**	**%**
**Temporary mental/emotional health disadvantages**		
Might make me bored or stir crazy	35	16.9
**Mental/emotional health disadvantages**		
Might lead to depression, anxiety or other mental health conditions	16	7.6
**Physical health disadvantages**		
Might lead to gaining weight, exercising less, or eating more	32	15.3
**COVID-19 disadvantages**		
Might not keep me from getting COVID	11	5.4
Might not reduce COVID-19 or keep my family from getting it	1	0.4
**Interpersonal disadvantages**		
Might miss interacting with friends and family	25	12.2
Miss getting out of the house to attend social events	15	7.3
**Financial disadvantages**		
Might lead to personal financial difficulties	7	3.3
Might weaken the economy	3	1.3
Might make me run out of or be low on supplies	8	3.8
**Occupational disadvantages**		
Might mean I lose my job	1	0.7
Might mean I will not be able to work	3	1.6

The percent of older adults mentioning each of 11 categories of facilitating circumstances is presented in Table 5. *Having things to do* was the most commonly elicited circumstance that might make it easier for participants to stay home (25.7%). This included things like watching movies/TV shows and doing yard work/other outside activities. Related to this, 6.5% mentioned *having the right weather* might make staying home easier. One in ten participants (10.8%) expressed that having *food and supplies delivered* to them might make it easier to stay home, and 5.5% mentioned *having access supplies* would help. In terms of interpersonal facilitators, 5.3% mentioned *living with someone* and 4.4% mentioned *being able to chat virtually*. An additional 4.0% mentioned *access to technology* as a facilitator.

**Table 5:** Frequency of perceived facilitators for staying home.

**…might make it easier for me to stay home**	**N**	**%**
**Financial facilitators**		
Having money	9	4.4
Getting financial assistance	3	1.2
**Occupational facilitators**		
Being able to work from home	3	1.4
Not having to work	4	2.2
**Leisure or recreational facilitators**		
Having things to do at home	53	25.7
**Interpersonal facilitators**		
Living with someone	11	5.3
Virtually chatting with others	9	4.4
**Supply chain facilitators**		
Having food and supplies delivered	22	10.8
Having supplies and access to buy supplies	11	5.5
Having access to technology	8	4.0
**Natural environment facilitators**		
Having the right weather	13	6.5
**Organizational and political environment facilitators**		
Having supportive government and state policies	5	2.5

## Discussion

This formative study tested factors associated with older adults' intention to stay home during the early part of the COVID-19 pandemic in the US. The means for intention and the four global constructs were positive, around four- on a five-point scale. Thus, for the most part, early in the epidemic, US older adults intended to stay home, saw it as a good thing to do, and believed they could stay home. However, there is room for improvement. Public health strategies are needed to help people continue to follow guidelines, particularly as we are required to stay home for longer periods.

The next finding of note was that when only demographic factors were included in the model, political ideology was the only demographic characteristic significantly associated with intention to stay home. Specifically, participants who were more conservative on the political spectrum reported lower intention to stay home. This is consistent with research (18) that also showed that older individuals who identified as strong Republicans reported lower intentions to follow social distancing orders compared to strong Democrats. This suggests that public health officials may need to tailor communications about staying home based on the political beliefs of the target audience. Also, we may need to look for and test for the possibility that communications might be interpreted differently depending on political ideology.

The primary aim of this study was to identify the beliefs underlying older adults' decision to stay home. Of note, when the four RAA global constructs were added to the regression model, the effect of political ideology on intention to stay home was no longer statistically significant. This points to the significant contributions of the global constructs in explaining the variation in intention to stay home, over and above the influence of several demographic factors. Indeed, the adjusted R^2^ of 0.732 in our model is higher than what was found in a study (16) of five COVID-19 intentions among UK adults. This implies that the RAA is a useful theory for research examining older adults' intention to stay home and a useful theory to be incorporated into interventions that encourage older adults to stay home. The next step is to identify which of these is the best target for an intervention (8). Given that there is room to improve intention and variation on each of the global constructs, the theory suggests that interventions address the constructs that make independent contributions to intention. In this study of older adults, injunctive norms and self-efficacy both showed statistically significant regression weights; attitude and descriptive norms did not. This implies that interventions prioritize injunctive norms and self-efficacy.

Injunctive norms represent people's perceptions about what people who are important to them think they should do. The significant effect of injunctive norms suggests that, in this sample of older adults, the influence of important people in their lives might be a key determinant of their intention to stay home. Therefore, health communication messages tailored for older adults should emphasize that people important to them are encouraging them to stay home. We did not conduct an elicitation to identify which social referents were important to these older adults for this behavior. However, other research (8) with the theory suggests that friends and family members are likely to be important social referents. Furthermore, research (22) documenting the relationship between trust in public health communication sources and following COVID-19 guidelines implies that public health professionals might be important social referents for this behavior in this population. Finally, the finding of political ideology as an associate of intention suggests that older adults might be paying attention to what political leaders say. Thus, it might be important for public health professionals to work with political leaders as opinion leaders.

Self-efficacy was the global construct most strongly associated with intention to stay home in this sample of older adults. This finding suggests that public health interventions should address older adults' confidence that they can stay home. There are two approaches to improving self-efficacy or capacity. One approach aims to address people's beliefs directly. Communication and educational campaigns can potentially help people see and come to believe that they have the capacity to stay home. Modeling is one effective way to improve self-efficacy (23). According to past research (24), modeling interventions should resemble the priority group, start with small steps, look to succeed but not immediately, and be reinforced for the behavior of staying home. Thus, these campaigns could include examples of how older people successfully overcame barriers and managed to stay home one step at a time. The second approach is to address the actual environment by removing barriers or adding facilitators at local, organizational, and governmental levels. Findings from our belief elicitation and other studies (25–27) suggest that interventions might want to prioritize older adults participating in meaningful but safe leisure and recreational activities and physical activity both inside and around their residences. Interventions can also include having a family member live with them, having home delivery of groceries, prescriptions, and other supplies, and having technology support to communicate with loved ones. Such interventions might help to improve the physical and mental health outcomes that older adults in our study were concerned about, although it is important to recognize that the interventions might not be equally accessible to all older adults.

The results of the salient belief elicitation reveal that following preventive behaviors like staying home involves perceptions about dimensions of wellness beyond just preventing COVID-19 infection. Most older adults are retired. Therefore, fewer of them are struggling with the financial and occupational disadvantages and barriers faced by those who are trying to work during the pandemic. However, the older adults in this study seem to be struggling with interpersonal, mental health, leisure, and other physical health issues. This is consistent with a study (28) that found one-quarter of their sample of adults 18 and older noted that their mental and physical health worsened since the start of the COVID-19 pandemic. Indeed, multidisciplinary mental health research and interventions are crucial to address during the pandemic (29). This suggests that interventions to help older adults stay home could help them find things to do at home as well as ways to connect safely with others. While addressing interpersonal and leisure facilitators may, on the surface, seem less essential than protecting people from exposure to a deadly disease, attending to health broadly defined could be critical to maintaining overall health as well as to helping people follow stay-at-home guidelines more consistently.

We also tested whether the relationships between the four RAA global constructs and intention to stay home were different for those age 65-71 vs. those age 72-94. The only evidence of a moderating effect was on the association between instrumental attitude and intention. Specifically, this association was statistically significant for the 65-71 year old group but not for the 72-94 year old group. This finding suggests that attitude could be an important focal point for interventions targeted at adults in their mid-sixties to early seventies. Addressing attitude could take the form of communication and education campaigns that present the advantages of staying home and address any potential negative consequences. To identify the specific advantages and disadvantages that might need to be addressed with messaging campaigns, we compared salient underlying advantageous and disadvantageous beliefs of the 65-71 group and the 72-94 group. We did not find statistically significant differences. However, we do want to highlight two differences that might suggest future research and intervention directions. First, the younger participants listed more concerns about gaining weight, exercising less, and eating more compared to the older participants (17.4% compared to 12.4%). This finding suggests providing younger old adults with opportunities to exercise in and around their residences, grocery delivery of healthy foods, and other weight control programs might help them to stay home. Second, more older participants listed missing interacting with family and friends than young-old participants (15.5 and 9.2%). Because older adults may have limited opportunities to freely interact with family/friends, providing these individuals with technology to virtually connect with others and providing them with safe ways to talk with family/friends in-person (e.g., both wear a mask, behind a plexiglass barrier) might address this potential disadvantage. Because this was an exploratory analysis, future research is needed to further examine these potential interactions.

A strength of this study is that the data are drawn from a nationally representative, probability sample resulting in generalizable findings. Furthermore, we were able to gather open-ended data about beliefs that elaborated on perceived advantages and disadvantages of staying home, as well as perceived facilitators of staying home. However, there are limitations that should be taken into account when interpreting the findings. First, this study used a cross-sectional design. It was designed to suggest potential factors and cannot be used to come to causal conclusions. Longitudinal and experimental studies are needed to assess the effects of COVID-19 mitigation interventions like stay-at-home orders on global constructs, intention, and behavior. Second, only five RAA items and three open-ended questions were included in the online survey due to resource constraints (e.g., time, funding). We assessed one of the two components of attitude (i.e., instrumental attitude but not experiential attitude) and one of the two components of perceived control (i.e., capacity, which we refer to as self-efficacy, but not the autonomy component). We asked only three of the six recommended open-ended questions. Thus, we could not draw conclusions based on the complete RAA theory. Third, on a related note, we used only one item per RAA construct. This limited our ability to assess the reliability of the measures. Fourth, the outcome assessed in this study was intention to engage in the desired behavior as opposed to the actual behavior of staying home. Thus, we did not test the intention-behavior relationship, a key part of the RAA. Finally, although it corresponded to guidelines, the behavior “stay in my house or apartment except to get food, care for a relative or friend, get necessary health care, go to an essential job, or exercise separated from others” is a complex one. It is possible that different participants interpreted the behavior differently. Future research with samples of older adults of color, for example, is needed to investigate and address health equity (30).

## Conclusions

Older adults are a priority population for increasing COVID protective behaviors. The current study provides insights into the global constructs associated with intention and the underlying beliefs of staying at home for older adults. Based on the findings in this study, we suggest three possible directions for public health interventions. The first is to develop interventions that enhance the self-efficacy of older adults to stay home to avoid unnecessary exposures. A second strategy supported by the current study's findings is to develop health communication messages that emphasize that people important to older adults think they should stay home. Third, for the youngest of the older adults, communication messages should emphasize the advantages of staying home. All of these strategies should keep in mind that the epidemic has wide ranging effects and that older adults are concerned with interpersonal, social, leisure, and mental health issues. These lessons can be applied to other behaviors, such as being vaccinated, as the COVID-19 pandemic persists, and we face future public health crises.

## Data Availability Statement

The raw data supporting the conclusions of this article will be made available by the authors, without undue reservation.

## Ethics Statement

The studies involving human participants were reviewed and approved by Indiana University Institutional Review Board. Written informed consent for participation was not required for this study in accordance with the national legislation and the institutional requirements.

## Author Contributions

JM and CO analyzed the data and JM, CO, and SM drafted the manuscript. JM, CO, KM, and SM contributed to conceptualization, investigation, methodology, and writing review and editing. All authors contributed to the article and approved the submitted version.

## Conflict of Interest

The authors declare that the research was conducted in the absence of any commercial or financial relationships that could be construed as a potential conflict of interest.
